# MicroRNAs Regulate Function in Atherosclerosis and Clinical Implications

**DOI:** 10.1155/2023/2561509

**Published:** 2023-08-29

**Authors:** Zhaoyi Li, Yidan Zhao, Sei Suguro, Rinkiko Suguro

**Affiliations:** ^1^State Key Laboratory of Quality Research in Chinese Medicine, Macau University of Science and Technology, Avenida Wai Long, Taipa, Macau SAR, China; ^2^Faculty of Medicine, School of Pharmacy, The Chinese University of Hong Kong, Shatin New Territories, Hong Kong SAR, China

## Abstract

**Background:**

Atherosclerosis is considered the most common cause of morbidity and mortality worldwide. Athermanous plaque formation is pathognomonic of atherosclerosis. The main feature of atherosclerosis is the formation of plaque, which is inseparable from endothelial cells, vascular smooth muscle cells, and macrophages. MicroRNAs, a small highly conserved noncoding ribonucleic acid (RNA) molecule, have multiple biological functions, such as regulating gene transcription, silencing target gene expression, and affecting protein translation. MicroRNAs also have various pharmacological activities, such as regulating cell proliferation, apoptosis, and metabolic processes. It is noteworthy that many studies in recent years have also proved that microRNAs play a role in atherosclerosis.

**Methods:**

To summarize the functions of microRNAs in atherosclerosis, we reviewed all relevant articles published in the PubMed database before June 2022, with keywords “atherosclerosis,” “microRNA,” “endothelial cells,” “vascular smooth muscle cells,” “macrophages,” and “cholesterol homeostasis,” briefly summarized a series of research progress on the function of microRNAs in endothelial cells, vascular smooth muscle cells, and macrophages and atherosclerosis. *Results and Conclusion*. In general, the expression levels of some microRNAs changed significantly in different stages of atherosclerosis pathogenesis; therefore, MicroRNAs may become new diagnostic biomarkers for atherosclerosis. In addition, microRNAs are also involved in the regulation of core processes such as endothelial dysfunction, plaque formation and stabilization, and cholesterol metabolism, which also suggests the great potential of microRNAs as a therapeutic target.

## 1. Introduction

According to statistics, as of 2019, an average of nearly 18 million people died of cardiovascular diseases every year, accounting for 32% of the global death toll. Atherosclerosis (AS) is the leading cause of death from cardiovascular diseases worldwide [1]. AS refers to a type of chronic blood disease in which a large amount of fat or cholesterol deposits on the walls of blood vessels and arteries, forming atherosclerotic plaques, resulting in reduced blood flow and blocked blood vessels. There are no obvious symptoms in the early stage of AS, but when the late symptoms do appear, the blood flow in the lumen is narrowed, and the blood flow is reduced, causing myocardial ischemia and hypoxia, and in severe cases, myocardial infarction, arrhythmia, and even sudden death [2]. The pathological mechanism of AS is complex, starting from the damaged intima, and the formation of plaques is also in the intima. In the early stage of the lesion, low-density lipoprotein (LDL) particles accumulate in the intima and are oxidized to oxidized LDL, causing an inflammatory response. Subsequently, proinflammatory monocytes bind to adhesion factors of endothelial cells, promoting the migration of monocytes to the arterial wall, where monocytes mature into macrophages in the intima. In the intima, macrophages transform into foam cells by engulfing lipoprotein particles, which further collect fat, cholesterol, and other substances. In the middle and late stages of the disease, the smooth muscle cells located in the media also enter the intima under the action of the medium produced by the aggregation of leukocytes, and proliferate, and accumulate in the arterial wall. As fat, cholesterol, and large amounts of cellular debris accumulate in the intima, plaques eventually form, which resemble “porridge.” With the increase of the plaque, some plaques are unstable and detached or are too large to split, causing blood vessel blockage and thrombus formation, which is also the main reason for the high mortality rate of AS [3]. Furthermore, the developmental, progression, and formation of clinically relevant atherosclerotic plaques consist of endothelial cells, vascular smooth muscle cells, and macrophages [4]. In short, vascular smooth muscle cells accumulate and aggregate in susceptible sites, resulting in dense cell arrangement and intima thickening, promoting endothelial cell activation and producing platelet-derived growth factor. With the stimulation of a large number of cytokines, the production of lipoproteins, proteoglycans, and fibronectin is promoted. As the retained lipoproteins are taken up by macrophages and vascular smooth muscle cells, lipid uptake and foam cell formation are enhanced, thereby accelerating the progression of the lesion [5].

**Figure 1:**
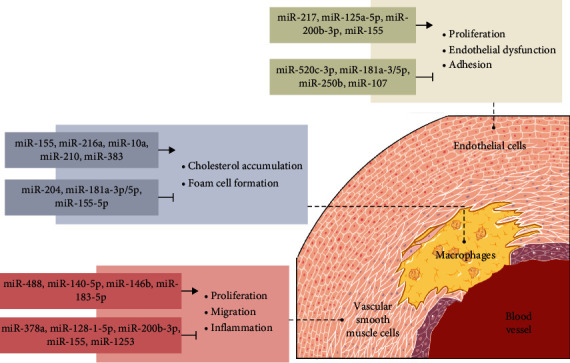
Regulation of microRNAs in different cells in AS.

MicroRNAs, also known as miRNAs or miRs, were first discovered in 1993 in Caenorhabditis *elegans* (*C. elegans*) [6, 7]. MicroRNAs are a class of small noncoding ribonucleic acid (RNA) regulatory molecules with a length of about 18–25 nucleotides, widely present in mammalian cells and body fluids, and involved in posttranscriptional regulation of gene expression and protein translation [8, 9]. Most mature microRNAs start from the initial primary transcription product primary microRNA (pri-miRNA), and pri-miRNA is cleaved by RNase III Drosha enzyme in the nucleus to become a single-stranded RNA precursor microRNA (pre-miRNA), and then the pre-miRNA is transported to the cytoplasm, where it is further processed into mature miRNA by RNase III Dicer [10]. Since the discovery of microRNAs, a variety of complex cellular events involved in it have been reported one after another [11]. For example, embryonic development [12], cell proliferation and apoptosis [13], and immune response [14], etc. At the same time, miRNAs are also involved in the regulation of many diseases, such as cancer [15], central nervous system diseases [16], infectious diseases [17], and diabetes [18]. Furthermore, microRNAs are required for the normal development of the cardiovascular system. MicroRNAs affect the development of AS by regulating the proliferation, adhesion, and endothelial dysfunction of endothelial cells, intervening in the accumulation of cholesterol mediated by macrophages and the formation of foam cells, and regulating the proliferation, migration, and inflammatory response of vascular smooth muscle cells (Figure 1) [7–9, 19–21].

Here, we systematically summarize the regulation mechanism of microRNAs on endothelial cells, vascular smooth muscle cells, macrophages, and cholesterol balance in the pathological process of AS and elaborate on the potential of microRNAs as clinical diagnostic biomarkers of AS, as well as future challenges, to provide a reference for researchers exploring related fields.

## 2. MicroRNAs Regulate Endothelial Cells

The abnormal function of endothelial cells is the initial stage of AS. Accumulating evidence indicates that endothelial dysfunction triggers an inflammatory response that drives the occurrence, development, and even rupture of atherosclerotic plaques and ultimately promotes the pathological development of AS [19, 22]. Some microRNAs have positive effects on improving AS. For instance, miR-520c-3p inhibits endothelial damage to attenuate AS by regulating vascular endothelial cell proliferation, apoptosis, and adhesion [23]. MiR-181a-5p, miR-181a-3p, and miR-250b have been proven to inhibit NF-*κ*B activation to prevent the occurrence of vascular inflammation, thereby slowing the progression of AS [24, 25]. Furthermore, miR-107 protects vascular endothelial cells against injury by inhibiting endoplasmic reticulum stress [26]. All in all, in endothelial cells, microRNAs mainly slow the progression of AS by inhibiting inflammatory responses, endoplasmic reticulum stress, or reducing endothelial cell damage. Conversely, some microRNAs promote the processes of AS. To give two examples of exacerbating AS, miR-217 has been shown to promote endothelial dysfunction by regulating the endothelial signaling center to trigger eNOS signaling, thereby aggravating AS [27]. MiR-125a-5p, miR-200b-3p, and miR-155 are thought to cause pyroptosis, apoptosis, and autophagy of endothelial cells, respectively, resulting in endothelial injury (Table 1) [19, 28, 29].

## 3. MicroRNAs Regulate Vascular Smooth Muscle Cells

Vascular smooth muscle cells reside in the medial layer of the arterial wall and are responsible for regulating vascular tone [30]. In AS, vascular smooth muscle cells migrate from the medial to the intima to proliferate and deposit in large numbers, further invading the plaque, causing the plaque to be unstable and prone to fall off, causing blood vessel blockage [21]. MicroRNAs have also been found to be involved in the transfer from endothelial cells to vascular smooth muscle cells, inhibiting apoptosis and inflammatory response or promoting cell proliferation and migration (Table 2) [31].

Some microRNAs inhibit the proliferation and migration of vascular smooth muscle cells and slow down the progression of AS. For example, miR-192-5p targets the expression of ATG7 and regulates autophagy [32]. MiR-214-3p acts by downregulating the expression of FOXO1 [33]. MiR-146b exerts its effect by downregulating the expression of Bag1 and MMP16 [34]. In addition, some microRNAs can also inhibit apoptosis and inflammatory responses, thereby alleviating AS. For instance, miR-17-5p has the effect of enhancing cell proliferation and repairing wounds and reduces apoptosis by upregulating SIRT7 expression and inhibiting p53 activation [35]. MiR-378a targeting IGF1 and TLR8 significantly inhibits inflammatory response [36]. MiR-128-1-5p inhibits the expression of inflammatory factors and apoptotic proteins by regulating the RMRP/miR-128-1-5P/Gadd45g signaling pathway [37]. Conversely, some microRNAs play the opposite role and accelerate AS deterioration. For example, both miR-183-5p and miR-488 can promote cell proliferation and migration by targeting myocyte enhancer factor 2C (MEF2B) [38, 39]. MiR-140-5p increases cell viability and promotes cell invasion by reducing the ROBO4 expression [40]. MiR-1253 binds to FOXF1 to inhibit its proliferation, thereby promoting apoptosis [41].

## 4. MicroRNAs Regulate Macrophages

Macrophages are an important part of the human immune system and can phagocytose and clear cell debris, dead cells, and pathogens *in vivo* [43]. During AS, on the one hand, macrophages are responsible for processing a large amount of cholesterol and triglycerides, and simultaneously help to clear some inflammatory substances [44]. On the other hand, once LDL enters, cholesterol accumulates in the blood vessel wall, and macrophages absorb the cholesterol oxidized by their free radicals, eventually turning into foam cells, which are conducive to plaque formation [45]. Recently, the roles of microRNAs in macrophages have been focused on, affecting the pathological process of AS by regulating inflammatory response, cholesterol metabolism, and foam cell formation (Table 3) [21, 45]. MiR-204 downregulates NFATc3 expression and prevents the formation of foam cells and AS [46]. MiR-181a-3p/5p and miR-155-5p attenuate inflammatory responses, and delay plaque formation, thereby slowing AS [24, 47]. Some microRNAs prevent AS by promoting mitochondrial oxidative metabolism, reducing ROS production and necroptosis, and improving cell survival, such as miR-10a [48], miR-210 [49], and miR-383 [49]. In addition, miR-368a promotes reverse cholesterol transport through the CD47-SIRP*α* axis and hinders AS progression [50]. However, miR-155 activates the NLRP3 inflammasome by regulating the ERK1/2 pathway and aggravates AS [51]. MiR-216a activates telomerase by regulating the Smad3/NF-*κ*B pathway and promotes AS development [50].

## 5. MicroRNAs Regulate Cholesterol Homeostasis

Cholesterol homeostasis is a key to lipid accumulation in atherosclerotic plaques and increases the risk and exacerbation of AS once the balance of cholesterol is disrupted [54]. In recent years, microRNAs have played key regulatory roles in lipid homeostasis and cholesterol homeostasis involved in AS development (Table 4) [44]. For example, miR-210-3p inhibited NF-*κ*B activation, reducing lipid accumulation and inflammatory responses [55]. MiR-34a, miR-33-5p, and miR-21 inhibit the development of AS by reducing intestinal cholesterol, regulating cholesterol efflux, and preventing foam cell formation, respectively [54, 56, 57]. In addition, miR-33a/b promotes lipid droplet accumulation by inhibiting apoptosis and accelerates AS [58]. So far, the role of microRNA in cholesterol homeostasis in AS needs more research support.

## 6. MicroRNAs Therapeutic Potential for AS

As summarized above, numerous studies have demonstrated the roles of microRNAs in regulating various pathological mechanisms in AS. MicroRNAs play important roles in the dysregulation that affects endothelial integrity, the function of vascular smooth muscle cells, macrophage, and cellular cholesterol homeostasis, which drives the initiation and growth of an atherosclerotic plaque [63]. In recent years, more studies have investigated the potential of microRNAs as therapeutic targets or biomarkers in AS (Table 5) [64]. For instance, hsa-miR-654-5p and hsa-miR-409-3p are the potentially critical biomarkers for AS patients [65]. Low expression of miR-211-5p and miR-675-3p are associated with the poor prognosis of AS [66, 67]. Low expression of miR-191-3p, miR-933, and miR-425-3p are related to the peripheral circulation of patients with lipid metabolism disorders, mainly LDL [68]. Moreover, dysregulation of microRNAs has a role in vascular aging [69]. Although there are few clinical studies of microRNAs for AS treatment, a variety of microRNAs have been found to reduce atherosclerosis in preclinical animal models, and some of these microRNAs have entered clinical studies in other diseases. For example, miR-494 is used to treat ischemic stroke (NCT03577093). miR-33 for the treatment of metabolic syndrome (NCT02606812) and heart failure (NCT02997462). miR-44 for the treatment of intracranial atherosclerosis (NCT03208166). miR-210 for the treatment of angina (NCT05374694). miR-155 for the treatment of bladder cancer (NCT03591367). miR-181 for the treatment of psoriasis (NCT05683769). miR-29 for the treatment of shoulder and neck pain (NCT02534558). These pieces of evidence fully illustrate the feasibility of microRNA therapy.

Although there are currently no microRNA drugs approved for the treatment of AS, drug candidates are in clinical development and clinical trials. Candidate drugs for microRNA therapy are mostly concentrated in antisense oligonucleotides (anti-miRs), microRNA mimics, and microRNA inhibitors. Among them, anti-miRs are the reverse complementary sequences of mature microRNAs, which bind to endogenous microRNAs and inactivate them through steric blocking, thereby regulating the function of microRNAs [70, 71], such as miR-494, miR-33, miR-712, and miR-114. MicroRNA mimics are synthetic double-stranded microRNAs fragments that regulate the post-translational function of microRNAs by specifically binding to target genes and inhibiting their transcription and translation [72], such as miR-210, miR-125a-5p, miR-29a-3p, miR -115, and miR-181a-3/5p. In addition, miRNA inhibitors are designed to have the reverse complementary strand of the target gene, and microRNAs affect the normal function of miRNA inhibitors by binding to the target site [73], such as miR-29 and miR-24-3p. Encouragingly, a handful of microRNA drug candidates have recently entered clinical trials. For example, MRG-110, as an antisense oligonucleotide, has entered phase I clinical trials (NCT03603431) to control angiogenesis and myocardial ischemia by targeting and inhibiting miR-92a [74]. Except for MRG-110, MRG-201 targets miR-29b to regulate the synthesis of extracellular matrix and has entered phase II clinical trials for fibrosis (NCT03601052) [75]. Furthermore, CDR132L improves cardiac systolic and diastolic functions by targeting miR-132 and has completed phase I clinical trials for the treatment of heart failure (NCT04045405) [76]. We summarized these microRNAs with AS clinical therapeutic potential in Table 6, and we also expect these microRNAs to achieve positive results in the clinical treatment of AS.

## 7. Discussion

As mentioned earlier, AS, as the most common cause of high morbidity and mortality in the world, is viewed as the result of four major steps, including (1) the initiation of endothelial cells activation and inflammation; (2) the promotion of intimal lipoprotein deposition, retention, modification, and foam cell formation; (3) the progression of complex plaques by plaque growth, enlargement of the necrotic core, fibrosis, thrombosis, and remodeling; (4) the precipitation of acute events such as myocardial infarction, unstable angina, ventricular fibrillation, or sudden coronary death [5]. As we have seen, microRNAs play indispensable roles in various stages of AS progression. Interestingly, the regulatory effects of most microRNAs can slow down the development of AS. For example, miR-520c-3p, miR-181a-5p, miR-181a-3p, miR-250b, and miR-107 have been shown to regulate endothelial cell proliferation, injury, or inflammatory responses, thereby reducing endothelial dysfunction [24, 27–29]. miR-204, miR-181a-3p/5p, miR-155-5p, miR-10a, miR-210, miR-383, miR-368a, miR-210-3p, miR-34a, miR-33-5p and miR-21 delays plaque formation by regulating cholesterol metabolism, avoiding conversion of macrophages into foam cells [32, 33, 35–37, 39]. miR-204, miR-181a-3p/5p, miR-155-5p, miR-10a, miR-210, miR-383, miR-368a, miR-210-3p, miR-34a, miR-33-5p, and miR-21 delays plaque formation by regulating cholesterol metabolism and avoiding conversion of macrophages into foam cells [24, 45, 48, 49, 51, 54, 55, 59, 60]. On the contrary, there are also a small number of microRNAs that can worsen the process of AS. For example, miR-217, miR-125a-5p, miR-200b-3p, and miR-155 cause damage to endothelial cells [20, 23, 25, 26]. miR-183-5p, miR-488, miR-140-5p, and miR-1253 accelerate intravascular plaque shedding [34, 40–42]. miR-155, miR-216a, and miR-33a/b promote cholesterol accumulation and accelerate foam cell and plaque formation [47, 49, 58]. Therefore, microRNA is very likely to be used as a potential biomarker in the clinical diagnosis of AS in addition to routine blood tests and imaging tests.

However, the role of microRNAs in AS is still being explored, and many questions still need to be answered to deepen our understanding. For example, the entry of exogenous microRNA may interfere with the normal regulatory mechanism in cells and cause side effects, adverse reactions, or immune responses [115–117], which may limit the therapeutic effect of microRNA and its safety issues. Additionally, since AS involves multiple cell types and signaling pathways, how to ensure that microRNAs target specific target cells or tissues to avoid affecting normal cells or tissues cannot be ignored. In this regard, many developers have tried using different delivery systems to improve the biodegradation and targeting of microRNAs *in vivo* [118]. For instance, Liu et al. [46] use nanodiamonds as delivery vehicles for microRNAs and implant them into induced pluripotent stem cells to promote the differentiation of induced pluripotent stem cells into cardiomyocytes and enhance the ability of damaged cardiomyocytes to recover cardiac function. Lolli et al. [119] use fibrin/hyaluronic acid (F/H) hydrogel to encapsulate microRNAs and implant subcutaneous damaged cartilage tissue and enhances the cartilage repair and regeneration function of endogenous cells. Based on nanoparticles and hydrogels, Li et al. [121] developed a new delivery system in which nanocarriers were encapsulated in injectable hydrogels, using this delivery system to deliver microRNAs to promote angiogenesis while reducing inflammation and effectively reduce the infarct size after myocardial infarction [122]. In addition, exosomes are another novel delivery system for microRNAs [123], which can improve the uptake of microRNAs by cells while promoting angiogenesis and wound healing [124]. However, the complex nanocarrier encapsulation process will inevitably cause off-target effects of microRNAs in vivo [125]. The charge properties of microRNAs affect their rate of release from hydrogels [120]. Treatment methods such as sonication and incubation with permeabilizers may cause exosomes to reorganize or deform and destroy the integrity of exosomes, thereby affecting the delivery efficiency of microRNAs [105, 124]. The solution to these problems is still a major research focus in the development of microRNAs delivery vectors in the future. Nonetheless, microRNAs are an exciting area of research because of their unique regulatory mechanisms and therapeutic potential in AS. It is hoped that continued clinical research and technological advances will shed light on these unresolved questions and advance the practical application of microRNAs as therapeutics for cardiovascular diseases.

## 8. Conclusions

MicroRNAs play important roles in AS development. We elucidate and summarize the recent studies on microRNAs regulation of the functions of endothelial cells, vascular smooth muscle cells, macrophages, and cholesterol metabolism in AS. MicroRNAs have the potential to be a novel diagnostic biomarker and therapeutic targets for atherosclerosis in the future.

## Figures and Tables

**Table 1:** The effects and mechanisms of microRNAs in regulating endothelial cells.

MicroRNAs	Effects	Mechanisms	References
miR-125a-5p	Promoted oxLDL-induced vascular endothelial cells pyroptosis	Inhibited TET2 expression, resulting in DNA methylation, mitochondrial dysfunction, increasing ROS production, and activated NF-*κ*B that induces activation of inflammasome and maturation, the release of proinflammatory cytokines	[28]

miR-181a-5p	Blocked blood vessel inflammation	Inhibited NF-*κ*B signaling pathway by targeting TAB2 protein	[24]

miR-181a-3p	Blocked blood vessel inflammation	Inhibited NF-*κ*B signaling pathway by targeting NEMO protein	[24]

miR-217	Promoted endothelial dysfunction	Regulated an endothelial signaling hub and downregulated a network of eNOS activators	[27]

miR-200b-3p	Promoted endothelial cells apoptosis	Promoted oxidative stress-induced cell apoptosis by targeting HDAC4	[29]

miR-107	Inhibited inflammatory response and endoplasmic reticulum stress of vascular endothelial cells	Regulated notch 1 signaling pathway	[26]

miR-520c-3p	Inhibited vascular endothelium dysfunction	Regulated AKT and NF-*κ*B signaling pathway by targeting RELA protein	[23]

miR-250b	Inhibited endothelial cells inflammation	Downregulated NF-*κ*B p65-ICAM1/VCAM1 axis	[25]

miR-155	Promoted endothelial cells autophagic activity	Inhibited Rheb-mediated mTOR/P70S6kinase/4EBP signaling pathway	[19]

OxLDL, oxidized low-density lipoprotein; TET2, tet methylcytidine dioxygenase 2; NF-*κ*B, nuclear factor *κ*-B; TAB2, mitogen-activated protein kinase 7-interacting protein 2; NEMO, NF-*κ*-B essential modulator; eNOS, endothelial nitric oxide synthase; HDAC4, histone deacetylase 4; RELA, nuclear factor NF-*κ*-B p65 subunit; ICAM1, intercellular adhesion molecule; VCAM1, vascular cell adhesion protein 1; mTOR, the mammalian target of rapamycin; 4EBP, translation initiation factor 4E-binding protein 1.

**Table 2:** The effects and mechanisms of microRNAs in regulating vascular smooth muscle cells.

MicroRNAs	Effects	Mechanisms	References
miR-192-5p	Inhibited cell proliferation and migration	Increased ATG7 expression	[32]

miR-183-5p	Promoted cell proliferation and migration	/	[39]

miR-17-5p	Inhibited cell proliferation and apoptosis	Increased SIRT7 expression	[35]

miR-378a	Inhibited cell proliferation, migration, and inflammation	Increased the expression IGF1 and TLR8 by PGC1*α*/NRF1/miR-378a regulatory axis	[36]

miR-214-3p	Eliminated cell proliferation, migration, and inflammation	Upregulated FOXO1 expression	[33]

miR-128-1-5p	Inhibited cell apoptosis and inflammation	Negatively regulated RMRP or Gadd45g	[37]

miR-326-3p	Inhibited cell viability, cell cycle distribution, and migration capacity	Inhibited VAMP3 expression	[42]

miR-1253	Inhibited proliferation and promotion of apoptosis	Inhibited FOXF1 expression	[41]

miR-146b	Inhibited cell proliferation and migration	Upregulated Bag1 and MMP16	[34]

miR-140-5p	Promoted cell viability, migration, and invasion	Reduced ROBO4 gene expression	[40]

miR-488	Promoted the proliferation and migrated ability	/	[38]

ATG7, autophagy related 7; SIRT7, sirtuin 7; IGF1, insulin-like growth factors 1; TLR8, Toll-like receptor 8; PGC1*α*, peroxisome proliferator-activated receptor gamma coactivator 1-*α*; NRF1, nuclear respiratory factor 1; FOXO1, forkhead box O1; RMRP, RNA component of mitochondrial RNA processing endoribonuclease; Gadd45g, growth arrest and DNA damage inducible gamma; VAMP3, vesicle associated membrane protein 3; FOXF1, forkhead box F1; Bag 1, bag cochaperone 1; MMP16, matrix metallopeptidase 16; ROBO4, roundabout guidance receptor 4.

**Table 3:** The effects and mechanisms of microRNAs in regulating macrophages.

MicroRNAs	Effects	Mechanisms	References
miR-204	Prevented foam cell formation	Upregulated NFATc3 to reduce SR-A and CD36 levels	[46, 52]

miR-181a-3p/5p	Delayed plaque formation	Reduced proinflammatory gene expression and macrophage infiltration	[24]

miR-155	Activated NLRP3 inflammasome	Blocking the ERK1/2 pathway	[51]

miR-10a	Promoted mitochondrial oxidative metabolism in macrophages	Promoted Dicer/miR-10a-dependent metabolic reprograming	[48]

miR-210	Reduced ROS production and necroptosis	Upregulated the HIF-1*α* level	[49]

miR-383	Reduced energy consumption and increased cell survival	Blocked the targeting of Parg protein	[49]

miR-155-5p	Mitigated vascular inflammation	Stimulated CTRP12 production	[47]

miR-368a	Promoted reverse cholesterol transport	Reduced SIRPA expression	[50]

miR-216a	Activated telomerase	Inhibited the Smad3/NF-*κ*B signaling pathway	[53]

NFATc3, nuclear factor of activated T cells 3; SR-A, the class A macrophage scavenger receptors; CD36, fatty acid translocase; NLRP3, NLR family pyrin domain containing 3; ERK1/2, extracellular signal-regulated protein kinase1/2; HIF-1*α*, hypoxia-inducible factor 1-*α*; CTRP12, C1q tumor necrosis factor-related protein 12; SIRPA, signal regulatory protein alpha; Smad3, suppressor of mothers against decapentaplegic 3; NF-*κ*B, nuclear factor *κ*B.

**Table 4:** The effects and mechanisms of microRNAs in regulating cholesterol homeostasis.

MicroRNAs	Effects	Mechanisms	References
miR-210-3p	Reduced lipid accumulation and inflammatory response	Inhibited IGF2/IGF2R to inhibit CD36 and NF-*κ*B expressions	[55]

miR-34a	Reduced intestinal cholesterol or fat absorption	Inhibited CYP7A1 and CYP8B1	[54]

miR-33a/b	Promoted lipid droplet accumulation	Inhibited apoptotic cell clearance via an autophagy-dependent mechanism	[58–62]

miR-33-5p	Regulated cholesterol efflux	Regulated the miR-33-5p/ABCA1/CS axis	[57]

miR-21	Influenced foam cell formation	Promoted p38-CHOP and JNK signaling pathway	[56]

IGF2, insulin-like growth factor 2; IGF2R, insulin-like growth factor 2 receptor; CD36, fatty acid translocase; NF-*κ*B, nuclear factor *κ*B; CYP7A1, cholesterol 7a-hydroxylase; CYP8B1, sterol-12a hydroxylase; ABCA1, ATP-binding cassette transporter A1; CS, citrate synthase; p38-CHOP, p38- C/EBP homologous protein; JNK, c-Jun N-terminal kinase.

**Table 5:** MicroRNAs as clinical biomarkers with therapeutic potential in the AS.

MicroRNAs	Cellular process	Cell type	Down- or upregulated	References
miR-654-3/5p	Apoptosis and inflammatory response	Endothelial cells	↓	[13, 77, 78]

miR-409-3p	Senescence	Endothelial cells	↑	[13, 79]

miR-933	Oxidative stress and inflammatory response	Endothelial cells	↑	[16, 80]

miR-122	Plaque stabilization	Endothelial cells	↑	[81, 82]

miR-92	Cholesterol buildup, inflammatory response	Endothelial cells	↓	[83]
	Foam cell formation	Macrophages		[84]

miR-211-5p	Inflammatory response	Macrophages	↓	[14, 85]

miR-675-3p	Adipogenesis and glucose metabolic	Macrophages	↓	[15, 86, 87]

miR-16	Inflammatory response	Macrophages	↓	[88, 89]

miR-155	Foam cell formation and cholesterol efflux	Macrophages	↓	[90]

miR-191-3p	Platelet activation and fibrous cap thinning	Smooth muscle cells	↓	[16, 63, 91]

miR-425-3/5p	Migration, phenotypic transformation, and proliferation	Vascular smooth muscle cells	↓	[16, 92]

miR-34	Vascular aging and inflammatory response	Vascular smooth muscle cells and endothelial cells	↑	[93, 94]
	Inflammatory response	Endothelial cells		[95]

miR-29	Vascular endothelial injury	Endothelial cells	↑	[96]
	Proliferation and migration	Vascular smooth muscle cells and endothelial cells		[97]

miR-21	Plaques vulnerability	Macrophages	↓	[98]
	Proliferation and migration	Vascular smooth muscle cells		[99]

**Table 6:** MicroRNAs therapeutic strategies in AS.

Strategy	MicroRNA	Target genes	Effects in AS	Clinical status	Reference
Antisense oligonucleotide (anti-miRs)	miR-494	Mef2A	Promoted plaque stabilization	Preclinical	[100]
	miR-33	ABCA1	Decreased lipid accumulation	Preclinical	[101]
	miR-712	TIMP3	Decreased endothelial inflammation	Preclinical	[102]
	miR-144	ABCA1/ABCG1	Regulated cholesterol metabolism and endothelial dysfunction	Preclinical	[103, 104]
	miR-92a	miR-92a	Regulated angiogenesis and ischemia	Phase Ⅰ	[74]
	miR-29b	miR-29b	Regulated extracellular matrix synthesis and fibrosis	Phase Ⅱ	[75]
	miR-132	miR-132	Improved cardiac function	Phase Ⅰ	[76]

microRNA mimics	miR-210	IGF2	Attenuated lipid accumulation and inflammation	Recruiting	[55, 105, 106]
	miR-125a-5p	Ninjurin 1	Attenuated vascular dysfunction	Preclinical	[107]
		CCL4	Decreased ox-LDL		[108]
	miR-29a-3p	TNFRSF1A	Suppressed proliferation, migration, and invasion of VSMCs	Preclinical	[109]
	miR-155	NLRP3	Attenuated inflammatory response	Preclinical	[7, 110]
	miR-181a-3p	NEMO	Inhibited vascular inflammation	Preclinical	[24, 111]
	miR-181a-5p	TAB2			

microRNA inhibitors	miR-29	LYPLA1	Promoted endothelial function	Preclinical	[112]
		CDC7	Regulated VSMCs proliferation and migration		[97]
	miR-24-3p	Bcl2L11	Prevented cell growth of VSMCs	Preclinical	[113]
		Imp*α*3	Inhibited the proliferation and migration of endothelial cells		[114]

Mef2A, myocyte enhancer factor 2A; ABCA1, ATP-binding cassette transporter A1; TIMP3, TIMP metallopeptidase inhibitor 3; ABCG1, ATP-binding cassette subfamily G member 1; IGF2, insulin like growth factor 2; Ninjurin 1, nerve injury-induced protein 1; CCL4, chemokine C-C-motif ligand 4; TNFRSF1A, tumor necrosis factor receptor superfamily member 1A; NLRP3, NLR family pyrin domain containing 3; NEMO, NF-*κ*-B essential modulator; TAB2, TGF-beta activated kinase 1-binding protein 2; LYPLA1, lysophospholipase 1; CDC7, cell division cycle 7; Bcl2L11, BCL-2 like protein 11; Imp*α*3, importin-*α*3; VSMCs, vascular smooth muscle cells; ox-LDL, oxidizied low-density lipoprotein.

## Data Availability

The data used to support the findings of this study are available from the corresponding author upon request.
